# Metabolic Heat Stress Adaption in Transition Cows: Differences in Macronutrient Oxidation between Late-Gestating and Early-Lactating German Holstein Dairy Cows

**DOI:** 10.1371/journal.pone.0125264

**Published:** 2015-05-04

**Authors:** Ole Lamp, Michael Derno, Winfried Otten, Manfred Mielenz, Gerd Nürnberg, Björn Kuhla

**Affiliations:** 1 Institute of Nutritional Physiology “Oskar Kellner”, Leibniz Institute for Farm Animal Biology (FBN), Dummerstorf, Germany; 2 Institute of Behavioural Physiology, Leibniz Institute for Farm Animal Biology (FBN), Dummerstorf, Germany; 3 Institute of Genetics and Biometry, Leibniz Institute for Farm Animal Biology (FBN), Dummerstorf, Germany; Institute of Zoology, CHINA

## Abstract

High ambient temperatures have severe adverse effects on biological functions of high-yielding dairy cows. The metabolic adaption to heat stress was examined in 14 German Holsteins transition cows assigned to two groups, one heat-stressed (HS) and one pair-fed (PF) at the level of HS. After 6 days of thermoneutrality and ad libitum feeding (P1), cows were challenged for 6 days (P2) by heat stress (temperature humidity index (THI) = 76) or thermoneutral pair-feeding in climatic chambers 3 weeks ante partum and again 3 weeks post-partum. On the sixth day of each period P1 or P2, oxidative metabolism was analyzed for 24 hours in open circuit respiration chambers. Water and feed intake, vital parameters and milk yield were recorded. Daily blood samples were analyzed for glucose, β-hydroxybutyric acid, non-esterified fatty acids, urea, creatinine, methyl histidine, adrenaline and noradrenaline. In general, heat stress caused marked effects on water homeorhesis with impairments of renal function and a strong adrenergic response accompanied with a prevalence of carbohydrate oxidation over fat catabolism. Heat-stressed cows extensively degraded tissue protein as reflected by the increase of plasma urea, creatinine and methyl histidine concentrations. However, the acute metabolic heat stress response in dry cows differed from early-lactating cows as the prepartal adipose tissue was not refractory to lipolytic, adrenergic stimuli, and the rate of amino acid oxidation was lower than in the postpartal stage. Together with the lower endogenous metabolic heat load, metabolic adaption in dry cows is indicative for a higher heat tolerance and the prioritization of the nutritional requirements of the fast-growing near-term fetus. These findings indicate that the development of future nutritional strategies for attenuating impairments of health and performance due to ambient heat requires the consideration of the physiological stage of dairy cows.

## Introduction

Ambient temperatures above 21°C cause serious heat stress in high-yielding dairy cows [1]. This leads to impairments of animal health, milk yield and reproduction [1,2], resulting in estimated economic losses of 897 million dollars in the USA, despite intensive cooling, every year [3]. In view of the higher frequency of extreme environmental heat events and increasing average temperatures predicted for the next decades [4], managing hot ambient temperatures will become an issue even for dairy farmers in areas of moderate climates [2,5]. For Germany, a recent report projected annual economic losses exceeding the 10%-margin for the national dairy farming sector [6].

As it will take time to genetically alleviate the heat stress susceptibility of the present Holstein dairy cattle, technical modification of environmental conditions (i.e. shading, fans, sprinklers etc.) and optimized feeding are crucial elements in the short-term management of the upcoming climatic challenges for dairy farmers to avoid severe production losses and maintain animal health.

Physiological reactions of high-yielding dairy cows to hot ambient temperatures and temperature humidity indexes (**THI**) above 68 to 72 include panting, elevated evaporation and increased rectal temperatures. Also serious depressions of feed intake and milk yield will occur [7].

Although the reduction in milk yield is mainly caused by a reduction of feed intake [8–10], the catabolism during heat stress is fundamentally different from catabolism induced by feed restriction at thermoneutrality. In thermoneutral starvation metabolism, cows rely on an extended oxidation of fatty acids from adipose tissues to maintain vital functions and spare glucose for lactose synthesis [11]. Thus, typical signs of starvation metabolism are increased plasma concentrations of non-esterified fatty acids (**NEFA**) and β-hydroxybutyric acid (**BHBA**) as well as reduced plasma glucose concentrations [11].

For heat stress metabolism during mid-lactation, there is no increase of NEFA concentrations [12,13] and adipose tissues are refractory to adrenergic lipolytic signals [14]. In contrast, the ratio of hepatic glucose output to milk lactose output is increased and the hepatic glucose release on catecholamines is preserved [14]. These recent studies propose a shift in metabolic utilization of macronutrients towards a preference of carbohydrate over lipid oxidation during heat stress. So far, these findings were mainly based on the analysis of blood plasma metabolites and endocrine reactivity in mid-lactating cows and disregarded the transition phase as a highly vulnerable moment.

Thus, we focused our research on the transition phase in which the cow undergoes a profound transformation from gestation to lactation accompanied by extensive metabolic adaptions [15]. Beyond the level of blood metabolite analysis, we wanted to examine the effective macronutrient oxidation by means of indirect calorimetry in an experiment involving Holstein cows in late gestation and again after parturition. In combination with blood metabolite analyses, this approach enabled novel insights in the different modes of adaptation of energy and nutrient metabolism in dairy cows experiencing heat stress at either one of two contrasting reproductive stages. Thereby, our results provide basic information on the design of precision feeding strategies alleviating heat load during hot weather conditions.

## Materials and Methods

All experimental procedures were in accordance with the German Animal Welfare Act (TierSchG) in its respective edition and were approved by the local Animal Research Committee (Landesamt für Landwirtschaft, Lebensmittelsicherheit und Fischerei (LALLF) of Mecklenburg-West Pommerania, Germany (LALLF M-V/TSD/7221.3–1.1-074/12)).

### Animals and Grouping

The experiment was performed with 14 German Holstein cows. Cows were genotyped for the HSP70.1 5′-UTR 895 [16]. Three different genotypes were identified and equally assigned to a heat-stressed (**HS**) or pair-fed (**PF**) group. Animals of each group were kept in one of two climatic chambers at the Tiertechnikum of the Leibniz Institute for Farm Animal Biology (FBN), Dummerstorf, Germany. All cows were at the end of their 2^nd^ parity (ante partum, **ap**), not milked within the -7 weeks prior to the expected calving date, and both groups were comparable in milk yield in the first lactation (data presented as mean ± standard error of mean) (7818.5 ± 22.0 kg), body weight (**BW**, 703.0 ± 24.6 kg ap, 577.1 ± 21.4 kg post-partum (**pp**); Table 1), and back fat thickness (**BFT**, 13.1 ± 1.4 mm ap, 9.1 ± 1.1 mm pp; Table 1) determined by ultrasound (Aloka SSD 500, PPG Hellige GmbH, Freiburg, Germany).

**Table 1:** Zootechnical data and energy balance of heat-stressed (HS) and pair-fed (PF) cows.

Period				
Item	Group	P1^1^	P2^1^	P-Value^2^
Body weight, kg	HSap	684.9 [588.5; 881]	670 [587.5; 864]	0.016
	PFap	724.2 [606; 796]	703.7 [604; 773.5]	0.031
	HSpp	569.9 [484.5; 716.5]	528.4 [463.5; 634.5]	0.031
	PFpp	584.3 [511; 694]	530.2 [449; 639.5]	0.031
Back fat thickness, mm	HSap	11.6 [6; 22]	11.6 [7; 21]	0.813
	PFap	15.0 [8; 23]	13.0 [7; 16]	0.313
	HSpp	9.8 [6; 19]	8.3 [5; 14]	0.313
	PFpp	8.5 [5; 13]	8.0 [5; 11]	0.438
Energy balance, MJ	HSap	-18.2 [-37.9; -1.2]	-50.3 [-69.3; -31.53]	0.016
	PFap	-16.5 [-24.4; -1.6]	-49.4 [-55.9; -38.0]	0.031

ap ante partum; pp post-partum; P1 period of thermoneutral conditions (THI = 59.7) with ad libitum feeding; P2 period of either pair feeding at thermoneutral (THI = 60.0) conditions (PF) or during ad libitum feeding under heat exposure (HS, THI = 76.1).

Numbers of animals analyzed per group: HSap n = 7, PFap n = 6, HSpp n = 6, PFpp n = 6.

^1^Values are given as mean [minimum; maximum].

^2^P-Values derived from Wilcoxon signed-rank test for paired samples.

There were no group differences between HS and PF at identical reproductive stages and experimental periods.

Both groups passed through a 13-day trial once in the ante partum stage (**HSap** and **PFap)** and again in the post-partum stage (**HSpp** and **PFpp).** Due to severe sickness of individual cows which were withdrawn from trial, groups amounted to: HSap n = 7, PFap n = 6, HSpp n = 6, PFpp n = 6 (S1 and S2 Tables).

### Experimental Procedure

The experiment was carried out from December 2012 to September 2013.

Prior to the experiment, animals were halter-trained and well adapted to the climatic chambers and respiration chambers with a light cycle ranging from 0600 to 1900 h, as described previously [17].

Before and after parturition, the 13 days-trial consisted of two 6-days periods **P1** and **P2** separated by one day of thermal transition. On day -21 ± 2.8 before actual parturition and again on day 22 ± 1.5 after parturition, cows were equipped with an indwelling jugular catheter (Certofix mono; B.Braun, Melsungen, Germany) and kept at thermoneutral conditions for 5 d in the climatic chamber and for another 24 h in an adjacent respiration chamber. Climatic conditions were identical for both HS and PF groups at constant 15°C and 63.3 ± 1.1% relative humidity (RH) resulting in a temperature-humidity-index (**THI**) [18] of 59.7 ± 0.1 with ad libitum feeding (experimental period P1). On the following transition day, cows were transferred back to the climatic chamber in which the air temperature (AT) was continuously increased to permanent 28°C for HS animals or left constant at 15°C for PF animals (experimental period P2). RH adjusted within 24 h to 52 ± 1.6% (THI = 76.1 ± 0.2) for HS and to 69.3 ± 1.7% (THI = 60 ± 0.3) for PF animals. THI was calculated from AT and RH according to NRC [18] as

THI=(1.8xAT(°C)+32)−(0.55−0.0055xRH(%))x(1.8xAT(°C)−26) (1)

On the transition day and the subsequent 6 days-period (experimental period P2), reduction of daily ad libitum feed intake of HS cows was calculated as percentage of the mean daily intake in P1 to provide the same relative amount of feed to PF cows during P2 [12]. Cows received a total mixed ration (**TMR**) twice daily (at 0700 h and 1500 h) in scale-connected troughs. Components and chemical analyses of TMR ap and pp are given in Table 2. Cows had free access to water which was warmed to 28°C for HS animals during P2. In the pp period, cows were milked at 0630 h and 1630 h. Feed intake, milk yield and water intake were recorded daily.

**Table 2:** Components and chemical analysis of TMR diets ante and post-partum.

Component	ante partum (Transition 1)	post-partum (Transition 2)
Ingredient [g/kg DM]		
Grass silage	180	223
Corn silage	438	352
Hay	62	44
Straw	62	0
Concentrate	247	367
Mineral feed	11	14
TMR DM [%]	45.0	48.2
Chemical analysis		
Crude protein [g/kg DM]	154.8	165.5
Crude fiber [g/kg DM]	189.0	159.6
Crude fat [g/kg DM]	27.8	35.8
Utilizable protein [g/kg DM]	147.2	152.7
Metabolizable energy [MJ/kg DM]	10.6	11.0

TMR total mixed ration; DM dry matter

### Vital Parameters

Rectal temperature, heart rate and respiratory rate were recorded twice a day before the morning and afternoon feeding to calculate the arithmetic daily mean. Rectal temperature was measured with a standard veterinary digital thermometer (MT1831, microlife AG, Widman, Switzerland). Heart rate was examined by auscultation with a stethoscope over 30 seconds. Respiratory rate was obtained by auscultation over the trachea for 60 seconds.

### Blood Sampling and Analyses

Before morning feeding, daily blood samples were collected during periods P1 and P2 into 9 ml-monovettes (Sarstedt, Nümbrecht, Germany) containing either ethylenediaminetetraacetate (**EDTA**), lithium heparin, or EDTA plus 50 μL of 1 M semicarbacide. Subsamples of blood were analyzed for hematocrit using EDTA containing glass capillaries (Sarstedt, Nümbrecht, Germany) which were centrifuged at 2,570 x g for 5 min. Blood in monovettes was centrifuged immediately after collection at 1,570 x g for 20 min at 4°C to obtain plasma which was stored at -80°C before analysis.

Plasma glucose, β-hydroxybutyric acid (**BHBA**), non-esterified fatty acids (**NEFA**) and urea were analyzed in EDTA plasma whereas creatinine was determined in lithium heparin plasma. Plasma concentrations were analyzed photometrically (Abx Pentra 400, Horiba, Kyoto, Japan) using kit NEFA-HR(2) (Wako Chemicals, Neuss, Germany) for NEFA, kits #RB 1008 and #LT-UR0010 (Labor+Technik Lehmann, Berlin, Germany) for BHBA and urea, respectively, and the kits #A11A01667 and #A11A01907 (Axon Lab, Reichenbach, Germany) for glucose and creatinine, respectively. Plasma 1-/3-methyl histidine was analyzed in EDTA plasma samples obtained on day 6 of periods P1 and P2 by high-pressure liquid chromatography (**HPLC**) techniques as described previously [19].

#### Analysis of Catecholamines

Concentrations of adrenaline (A) and noradrenaline (NA) were analyzed from semicarbacide treated plasma samples obtained between day 2 and 5 of periods P1 and P2 by HPLC with electrochemical detection after extraction from plasma by absorption on aluminum oxide [20]. Intra- and interassay coefficients of variation, tested on midrange samples, were 4.6% and 8.5% for epinephrine and 3.3% and 1.9% for norepinephrine.

### Indirect Calorimetry

In the evening of the 5^th^ day of periods P1 and P2, animals were transferred to an open-circuit respiration chambers after measurement of BW [17]. On day 6 of period P1 and P2, concentrations of CO_2_ and CH_4_ in the chamber were analyzed by infrared-absorption and the concentration of O_2_ paramagnetically (SIDOR, SICK MAIHAK GmbH, Reute, Germany) every 6 min. Based on the measurements of O_2_ consumption and CO_2_ and CH_4_ production, daily heat production (**HP**) was calculated according to Brouwer [21] and given as a ratio per metabolic weight **(mBW)**:

$$\text{HP}/\text{mBW}\left(\text{kJ}/{\text{kg}}^{0.75}\right)=\left(16.18{\text{V}}_{\text{O}2}\left(\text{L}\right)+5.02{\text{V}}_{\text{CO}2}\left(\text{L}\right)-2.17{\text{V}}_{\text{CH}4}\left(\text{L}\right)-5.99{\text{N}}_{\text{u}}\left(\text{g}\right)\right)/\text{mBW}\left({\text{kg}}^{0.75}\right)$$ (2)

Because total CO_2_ production (**V**_**CO2**_) measured is the sum of fermentative (**CO**_**2 ferm.**_) and metabolic CO_2_ (**CO**_**2 metab.**_). **CO**_**2 ferm.**_ was estimated according to Chwalibog et al. [22]:

$${\text{V}}_{\text{CO}2\;\text{ferm}.}\;\left(\text{L}\right)=1.7\;\text{x}\;{\text{V}}_{\text{CH}4}\;\left(\text{L}\right)$$ (3),

in which the factor 1.7 is constant for a variety of diet compositions [23]. Accordingly, V_**CO2 metab.**_ was calculated by subtracting V_CO2 ferm._ from V_CO2_. Net carbohydrate oxidation (**COX**) and net fat oxidation (**FOX**) were calculated by the following equations described recently [24] and given as a ratio per mBW:

$$\mathrm{FOX}/\mathrm{mBW}\left(g/kg^{0.75}\right)=(1.69V_{O2}\left(L\right)-1.69V_{\mathrm{CO}2\mathrm{metab}.}\left(L\right)-2.03N_{u}\left(g\right))/\mathrm{mBW}\left(kg^{0.75}\right)$$ (4)

$$\text{COX}\;/\;\text{mBW}\;\left(\text{g}\;/\;{\text{kg}}^{0.75}\right)=(4.75\;{\text{V}}_{\text{CO}2\;\text{metab}.}\;\left(\text{L}\right)-3.23\;{\text{V}}_{\text{O}2}\;\left(\text{L}\right)-2.60\;{\text{N}}_{\text{u}}\;\left(\text{g}\right))\;/\;\text{mBW}\;\left({\text{kg}}^{0.75}\right)$$ (5)

Urine N excretion (**N**_**u**_) was not measured and set to zero, thus accepting an error of about 10% [25] in the absolute values of HP, COX and FOX.

Exhausted air from each respiration chamber was chilled to 5°C to quantify the resulting water condensate collected for 24 hours. Conductance (**C**) was calculated from HP, average rectal temperature (**RT**), average ambient temperature (**AT**) and mBW based on an equation published by Klaus et al. [26]:

$$\text{C}\left(\text{kJ}\;/\;\left(\text{K}*{\text{kg}}^{0.75}\right)\right)=\text{HP}\;\left(\text{kJ}\right)\;/\;\left(\left(\text{RT}\;\left({}^{\circ}\text{C}\right)-\text{AT}\;\left({}^{\circ}\text{C}\right)\right)*\text{mBW}\right)$$ (6)

Metabolizable energy (**ME**) intake was calculated according to the recommendations of the German Society of Nutrition Physiology [27]:

$$\begin{array}{l} \text{ME}\;\left(\text{MJ}\;/\;\text{kg DM}\right)=6.0756+0.19123\;\text{EE}\;\left(\text{g}\;/\;\text{kg}\right)+0.02459\;\text{CP}\;\left(\text{g}\;/\;\text{kg}\right)\text{-}0.000038\;{\text{CF}}^{2}\;\left({\text{g}}^{2}\;/\;{\text{kg}}^{2}\right)\\ -0.002139\;{\text{EE}}^{2}\;\left({\text{g}}^{2}\;/\;{\text{kg}}^{2}\right)-0.00006\;{\text{CP}}^{2}\;\left({\text{g}}^{2}\;/\;{\text{kg}}^{2}\right) \end{array}$$ (7)

in which **CP** is crude protein, **CF** is crude fiber, and **EE** is crude fat from ether extract. Exclusively for ap animals, energy balance was calculated by subtracting measured HP from ME intake.

### Statistical Analysis

Repeated measures data (DMI, vital parameters and plasma concentrations excluding 1-/3-methyl histidine) were analyzed for effects of group, day, and their interaction during period 2 as a completely randomized design using PROC MIXED with a repeated measurements analysis with day as the repeated effect. Data were tested to determine the structure of best fit with either an auto-regressive or a compound-symmetric covariance structure according to the lower Akaike information criterion (AIC). Before analysis, these data were baseline-corrected to the mean value of the last days of thermoneutral P1 conditions varying due to analytical restrictions. Namely, these were for hematocrit and plasma metabolites days 5–6 of P1 and the transition day, for DMI and vital parameters days 4–6 of P1 and for A and NA days 3–5 of P1. For the comparison of group differences on a daily basis, a Tukey-Kramer test was applied and results are plotted in S1, S2, S3 and S4 Figs.

BW, BFT and plasma 1-/3-methyl histidine concentrations were obtained only once on day 6 of each period and evaluated as single-point measurements. Data acquired during indirect calorimetry, were analyzed as sums of the 24-hours data acquisition. Non-parametric methods were assigned for statistical testing of AUC or single measurements as well as the mean values of the last days of P1 used for baseline correction of continuously measured variables. Differences between periods P1 and P2 in the same group were analyzed using the Wilcoxon signed rank sum test included in the UNIVARIATE procedure of SAS. Analysis of differences between two groups of the same period and the same reproductive stage were performed using the exact Wilcoxon-Mann-Whitney test of the NPAR1WAY procedure. We did not test for differences between groups before versus after parturition due to changes in the composition of groups from the ap to pp stage, except for rectal temperatures. Results of all tests performed were considered significant if P<0.05, and as a trend if 0.05≤P<0.07.

Values of 24-hours sums and single measurements are given as mean [minimum; maximum]. Repeated measurements are given as daily means ± standard error. All statistical analyses were performed using SAS software package (version 9.4, SAS Institute Inc., Cary, NC, USA).

## Results

### Intake of forage and water is affected by environmental heat in both reproductive stages

**Fig 1:**
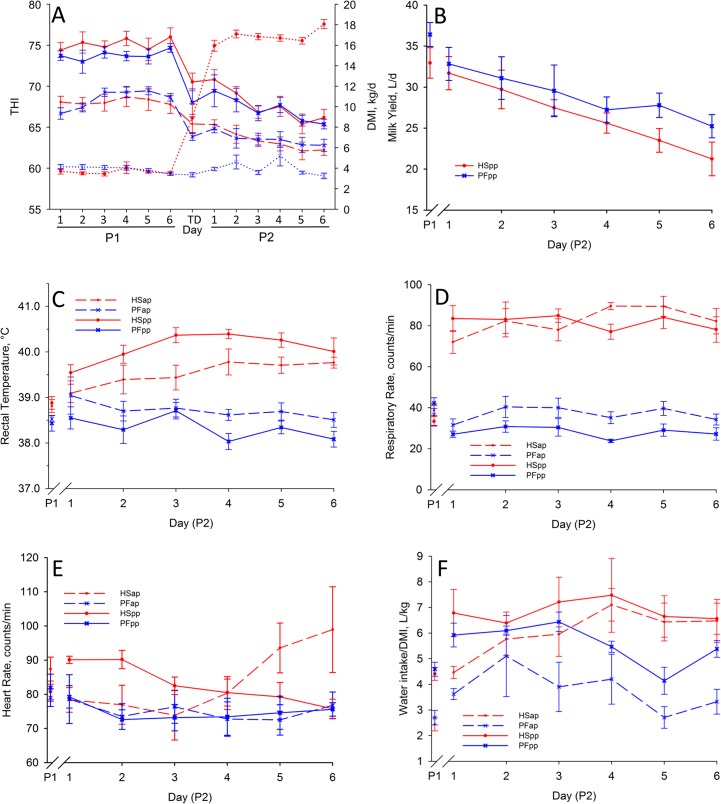
Nutritional and Vital Data. (A) Temperature humidity index (THI, solid or dashed lines), and dry matter intake (DMI; dotted lines; given as mean of both heat-stressed (HS, red) and pair-fed groups (PF, blue), respectively) during the six days of P1 and P2 and the transition day (TD). Effects of HS and PF on (B) daily milk yield, (C) rectal temperatures, (D) respiratory rate, (E) heart rate and (F) Water intake/DMI. In experimental period 1 (P1) all animals were kept at thermoneutral conditions (THI = 59.7) with ad-libitum feeding for six days. During period 2 (P2), HS cows (red lines) were heat-stressed (THI = 76.1), whereas PF cows (blue lines) were pair-fed in thermoneutrality (THI = 60.0) for six days, once ante partum (ap, dashed lines) and again post-partum (pp, solid lines). All data given as mean ± SEM; for P1 the mean of the last days of period 1 is given in Fig 1B-F as used for baseline correction; numbers of animals analyzed per group: HSap n = 7, PFap n = 6, HSpp n = 6, PFpp n = 6.

Heat stress reduced dry matter intake (**DMI**) in P2 to an average of 62.8% and 61.9% of the ad libitum DMI in P1 for the ap and pp stage, respectively (day: P < 0.05, Fig 1A, Table 3). By design, feed offered to PF animals during P2 was restricted by the extent the HS cows reduced their voluntary DMI in P2, resulting in a mean DMI of 62.3% relative to P1 for PF cows in the ap and pp stage (Fig 1A, Table 3). The reduction of DMI led to a general decrease of BW by a mean of 4.3% for HS and 6.1% for PF animals for both stages (P < 0.05, Table 1). Until day 6 of P2 (nadir), milk yield in HSpp and PFpp declined by -35.9% ± 4.5 or -30.6% ± 3.1 compared to the last days of P1, respectively (day: P < 0.05, Fig 1B). We did not observe changes in BFT for both groups during P1 and P2, neither before nor after parturition. There were no group differences in terms of BW, BFT (P > 0.05, Table 1) and milk yield (group: P > 0.05, Fig 1B, Table 3). As compared to P1, HS cows responded to environmental heat by an increased rectal temperature (ap: group*day: P < 0.05; pp: group, day, group*day: P < 0.05, Fig 1C, Table 3) and respiratory rate (group: P < 0.05, Fig 1D, Table 3), but significant alterations of heart rate were only apparent in the pp stage (group: P < 0.05, Fig 1E, Table 3). Rectal temperatures during heat stress were higher in HSpp cows than in the HSap group (group, day: P < 0.05). The mean respiratory rate during the last days of P1 was higher in PFpp than in HSpp (P < 0.05), which was the only significant difference of the P1 means used for baseline correction. Water intake per DMI was also increased from P1 to P2 in HS during both, the ap and pp stage but not under PF conditions (group: P < 0.05, Fig 1F, Table 3).

**Table 3:** Effects of heat stress (HS) or pair-feeding (PF) on nutritional, vital and metabolic variables.

		P1		P2		Covariance	P-Value		
Item	Stage	HS	PF	HS	PF	structure	Group	Day	Group*Day
DMI, kg	ap	10.63 ±0.93	11.3 ±0.27	6.67 ±0.69	6.82 ±0.72	AR	0.557	0.021	0.957
	pp	16.35 ±0.86	15.19 ±0.42	10.12 ±0.85	9.78 ±0.89	AR	0.515	<0.001	0.795
Milk yield, L	pp	32.95 ±1.86	36.42 ±1.47	26.54 ±1.68	28.96 ±2.04	AR	0.476	<0.001	0.648
Water intake/DMI, L/kg	ap	2.44 ±0.26	2.7 ±0.28	6.03 ±0.66	3.81 ±0.77	CS	0.034	0.108	0.064
	pp	4.41 ±0.26	4.59 ±0.27	6.85 ±0.86	5.58 ±0.35	AR	0.010	0.221	0.517
Rectal temperature, °C	ap	38.81 ±0.14	38.53 ±0.07	39.53 ±0.23	38.72 ±0.21	AR	0.081	0.958	0.030
	pp	38.88 ±0.14	38.43 ±0.17	40.09 ±0.18	38.33 ±0.2	CS	0.000	0.033	0.005
Heart rate, counts/minute	ap	87.38 ±3.5	79 ±2.58	83.66 ±6.87	75.07 ±4.86	AR	0.647	0.136	0.103
	pp	80.75 ±2.21	81.94 ±3.96	83.04 ±2.88	74.76 ±3.7	AR	0.020	0.095	0.091
Respiratory rate, counts/minute	ap	38.7 ±2.51	36.67 ±5.27	82.26 ±4.99	36.85 ±3.63	AR	<0.001	0.077	0.433
	pp	33.36 ±2.35	42.28 ±2.58	81.81 ±5.57	28.08 ±2.56	AR	<0.001	0.098	0.971
Hematocrit, %	ap	34.05 ±0.82	33.5 ±1.14	32.35 ±0.73	32.28 ±1.05	AR	0.234	0.021	0.422
	pp	31.28 ±0.91	28.89 ±0.86	28.19 ±1.18	29.17 ±0.91	CS	0.009	0.001	0.004
Glucose, mmol/L	ap	3.94 ±0.15	3.56 ±0.08	3.63 ±0.05	3.27 ±0.15	CS	0.912	<0.001	0.016
	pp	3.31 ±0.26	3.09 ±0.11	2.82 ±0.24	2.49 ±0.22	AR	0.552	0.002	0.656
BHBA, mmol/L	ap	0.48 ±0.04	0.47 ±0.03	0.59 ±0.04	0.57 ±0.12	CS	0.979	0.038	0.594
	pp	1.18 ±0.25	0.94 ±0.19	1.85 ±0.55	2.04 ±0.45	AR	0.384	0.004	0.922
NEFA, μmol/L	ap	182.29 ±30.65	179.44 ±23.2	506.79 ±77.62	603.83 ±140.57	AR	0.435	0.002	0.600
	pp	749.06 ±118.42	720.17 ±119.91	856.39 ±127.43	1205.75 ±185.39	AR	0.016	<0.001	0.165
Urea, mmol/L	ap	3.88 ±0.39	2.86 ±0.28	3.78 ±0.36	3.07 ±0.26	AR	0.059	0.060	0.981
	pp	3.59 ±0.38	3.15 ±0.14	5.18 ±0.66	3.57 ±0.22	AR	0.027	0.061	0.461
Creatinine, μmol/L	ap	93.78 ±4.02	91.39 ±5.67	113.87 ±5.12	97.92 ±5.27	AR	0.001	0.022	0.023
	pp	80.3 ±4.38	77.27 ±3.13	104.44 ±7.91	75.97 ±2.78	CS	0.001	0.007	0.021
Urea/Creatinine-Ratio, mmol/μmol	ap	0.0417 ±0.0042	0.0323 ±0.0043	0.0336 ±0.0031	0.0310 ±0.0024	CS	0.022	<0.001	0.187
	pp	0.0445 ±0.0039	0.0411 ±0.0021	0.0494 ±0.0057	0.0471 ±0.004	AR	0.702	0.429	0.452
Noradrenaline, pg/mL	ap	199.33 ±25.7	250.68 ±28.61	252.37 ±32.89	204.26 ±24.61	AR	0.011	0.566	0.183
	pp	178.32 ±20.47	228.4 ±40.52	255.4 ±34.61	227.13 ±47.77	AR	0.079	0.302	0.271
Adrenaline, pg/mL	ap	71.16 ±4.27	97.36 ±10.33	89.04 ±10.83	89.63 ±16.04	CS	0.119	0.277	0.508
	pp	80.18 ±5.6	64.29 ±8.28	104.79 ±11.42	52.62 ±6.29	CS	0.008	0.510	0.066

ap ante partum; pp post-partum; P1 period of thermoneutral conditions (THI = 59.7) with ad libitum feeding; P2 period of either pair feeding at thermoneutral (THI = 60.0) conditions (PF) or during ad libitum feeding under heat exposure (HS, THI = 76.1); values given as mean ±standard error of mean; AR autoregressive; CS compound symmetric.

Numbers of animals analyzed per group: HSap n = 7, PF ap n = 6, HS pp n = 6, PF pp n = 6.

### Environmental heat causes marked alterations in hematocrit and plasma metabolite concentrations

**Fig 2:**
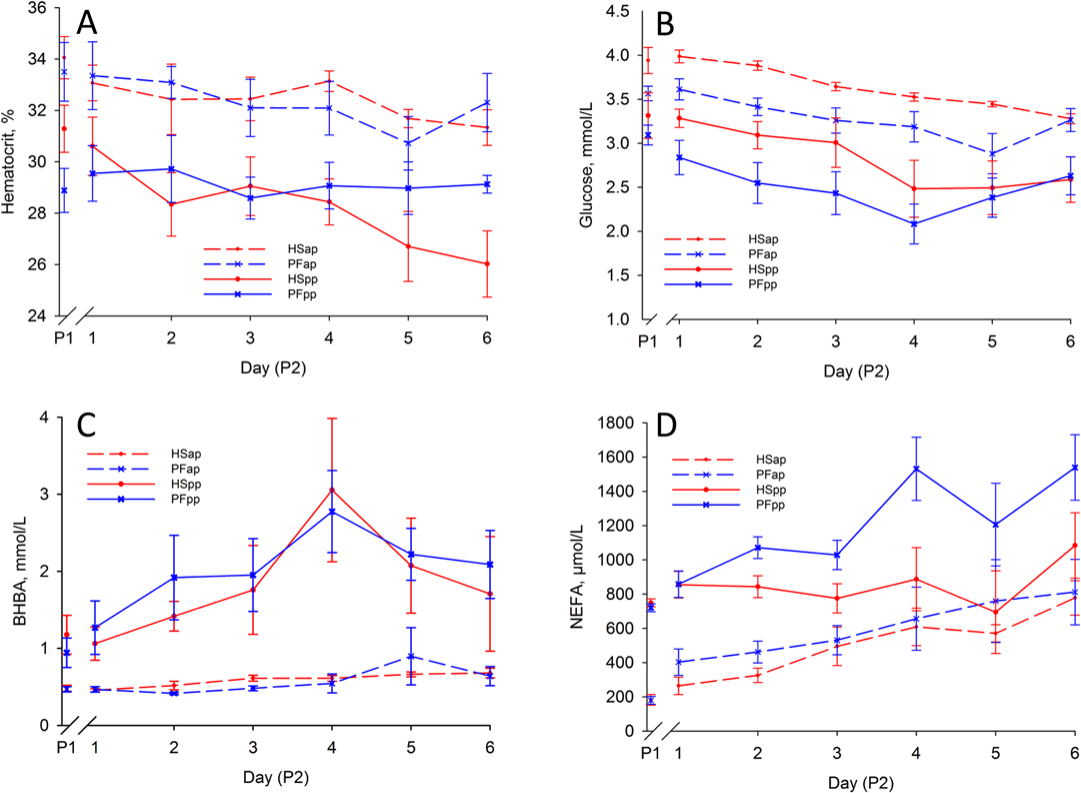
Hematocrit and Plasma Metabolites. Effect of heat stress (HS) and pair-feeding (PF) on (A) hematocrit, (B) plasma glucose, (C) plasma β-hydroxybutyric acid (BHBA), (D) plasma non-esterified fatty acids. In experimental period 1 (P1) all animals were kept at thermoneutral conditions (THI = 59.7) with ad-libitum feeding for six days. During period 2 (P2), HS cows (red lines) were heat-stressed (THI = 76.1), whereas PF cows (blue lines) were pair-fed in thermoneutrality (THI = 60.0) for six days, once ante partum (ap, dashed lines) and again post-partum (pp, solid lines). All data given as mean ± SEM; for P1 the mean of the last days of period 1 is given as used for baseline correction. BHBA: β-hydroxybutyric acid; NEFA: non-esterified fatty acids. Numbers of animals analyzed per group: HSap n = 7, PFap n = 6, HSpp n = 6, PFpp n = 6.

**Fig 3:**
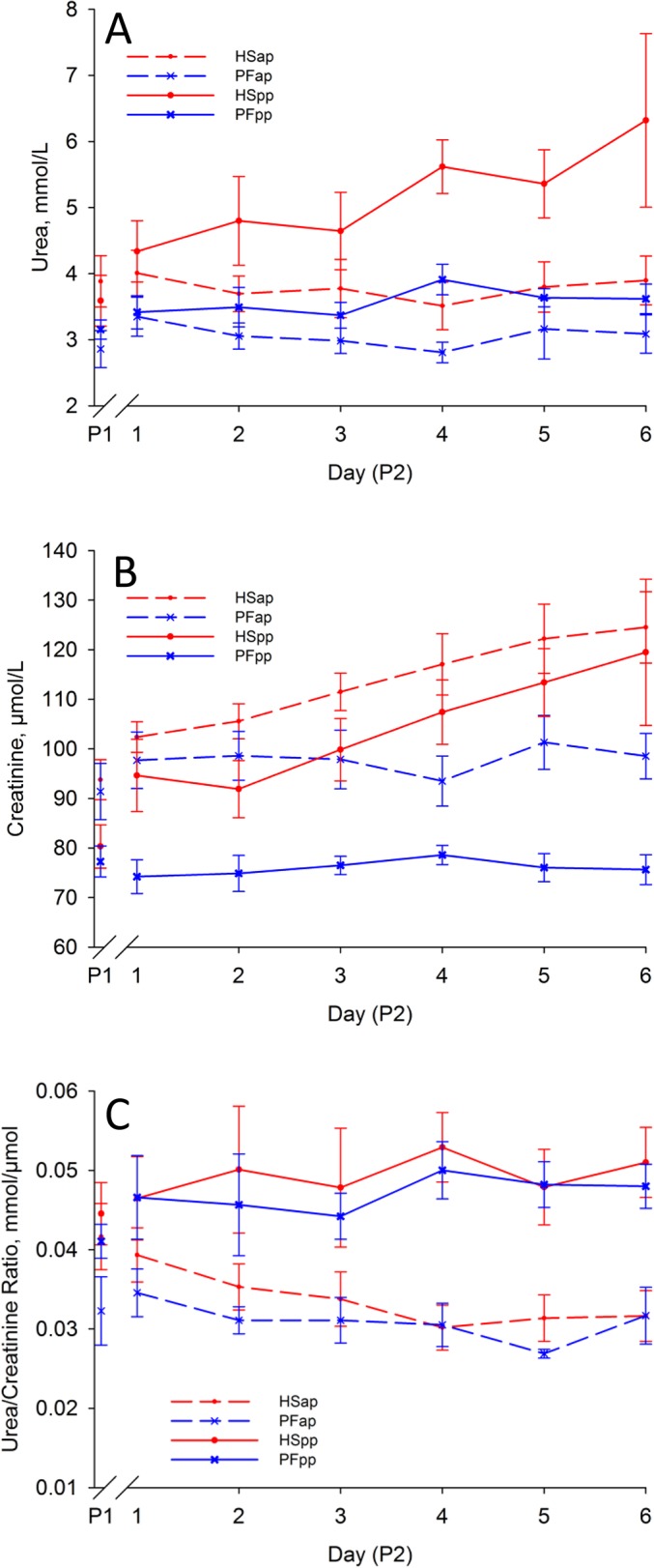
Plasma Urea and Creatinine. Effect of heat stress (HS) and pair-feeding (PF) on (A) plasma urea, (B) plasma creatinine, (C) urea/creatinine ratio. In experimental period 1 (P1) all animals were kept at thermoneutral conditions (THI = 59.7) with ad-libitum feeding for six days. During period 2 (P2), HS cows (red lines) were heat-stressed (THI = 76.1), whereas PF cows (blue lines) were pair-fed in thermoneutrality (THI = 60.0) for six days, once ante partum (ap, dashed lines) and again post-partum (pp, solid lines). All data given as mean ± SEM; for P1 the mean of the last days of period 1 is given as used for baseline correction. Numbers of animals analyzed per group: HSap n = 7, PFap n = 6, HSpp n = 6, PFpp n = 6.

Analyses of daily blood samples revealed a marked decrease of hematocrit in early-lactating cows under HS conditions from P1 to P2 (group, day, group*day: P < 0.05), but not in PFpp controls (Fig 2A, Table 3). Whereas in late-gestating cows, hematocrit decreased slightly during P2 in both groups (time: P < 0.05). Glucose concentrations decreased in all groups during P2 (time: P < 0.05, Fig 2B, Table 3), whereas BHBA concentrations increased in all groups compared to P1 (day: P < 0.05, Fig 2C). NEFA concentrations increased from P1 to P2 in all groups with the exception of HSpp, which showed almost constant NEFA concentrations (ap: day: P < 0.05, pp: group, day: P < 0.05, Fig 2D, Table 3). Plasma urea concentrations increased more under heat-stress than under PF conditions in early-lactating cows (group: P < 0.05, day: P < 0.07). In contrast, urea concentrations showed minor changes in late-gestating cows (group, time: P < 0.07, Fig 3A, Table 3). Heat stress increased creatinine concentrations in both stages (group, day, group*day: P < 0.05, Fig 3B, Table 3). During P2, the urea/creatinine ratio did not change in early-lactating cows (P > 0.05) and PFap, but decreased in HSap (group, day: P < 0.05, Fig 3C, Table 3).

### The metabolic shift towards fat oxidation during pair-feeding is absent under high ambient temperatures

**Fig 4:**
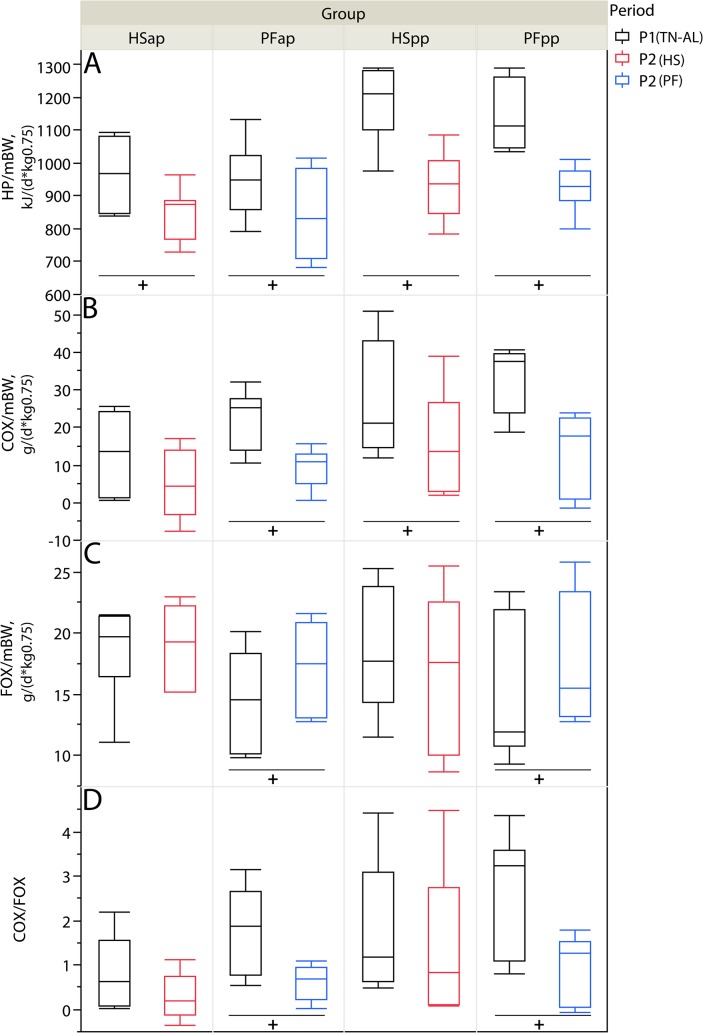
Indirect Calorimetry. Effect of heat stress (HS) and pair-feeding (PF) on (A) heat production per metabolic weight (HP/mBW), (B) net carbohydrate oxidation per metabolic weight (COX/mBW), (C) net fat oxidation per metabolic weight (FOX/mBW), (D) COX/FOX ratio. In experimental period 1 (P1, black) all animals were kept at thermoneutral conditions (TN, THI = 59.7) with ad-libitum feeding (AL) for six days. During period 2 (P2), HS cows (red) were heat-stressed (THI = 76.1), whereas PF cows (blue) were pair-fed in thermoneutrality (THI = 60.0) for six days, once ante partum (ap) and again post-partum (pp). Cows underwent a 24-hours indirect calorimetry analysis on day 5 of each period. Numbers of animals analyzed per group: HSap n = 7, PFap n = 6, HSpp n = 6, PFpp n = 6; all data given as sums of continuous measurements over 24 hours on day 6 of each period; ^**+**^P < 0.05 in Wilcoxon signed rank sum test for paired samples of the same group; there were no group differences between independent samples of the same reproductive stage and period according to the exact Wilcoxon-Mann-Whitney test (P > 0.05).

Indirect calorimetry revealed that daily heat production per metabolic weight (HP/mBW) is reduced under PF as well as under HS conditions (P < 0.05, Fig 4A). In all groups except the HSap, this is paralleled by a reduction of COX/mBW from P1 to P2 (P < 0.05, Fig 4B). Simultaneously, FOX/mBW increased in PF during P2 ante and post-partum (P < 0.05), whereas FOX/mBW was not altered under high ambient temperatures (P > 0.05, Fig 4C). The COX/FOX ratio as an indicator of energy source preference was lowered from P1 to P2 in PF animals of both stages (P < 0.05), but remained constant under HS conditions (Fig 4D).

The energy balance was negative for all animals before parturition in P1, and both HSap and PFap showed a similar intensification of this state from P1 to P2 (P < 0.05, Table 1).

### Conductance and evaporative water emission are increased under high ambient temperature

**Fig 5:**
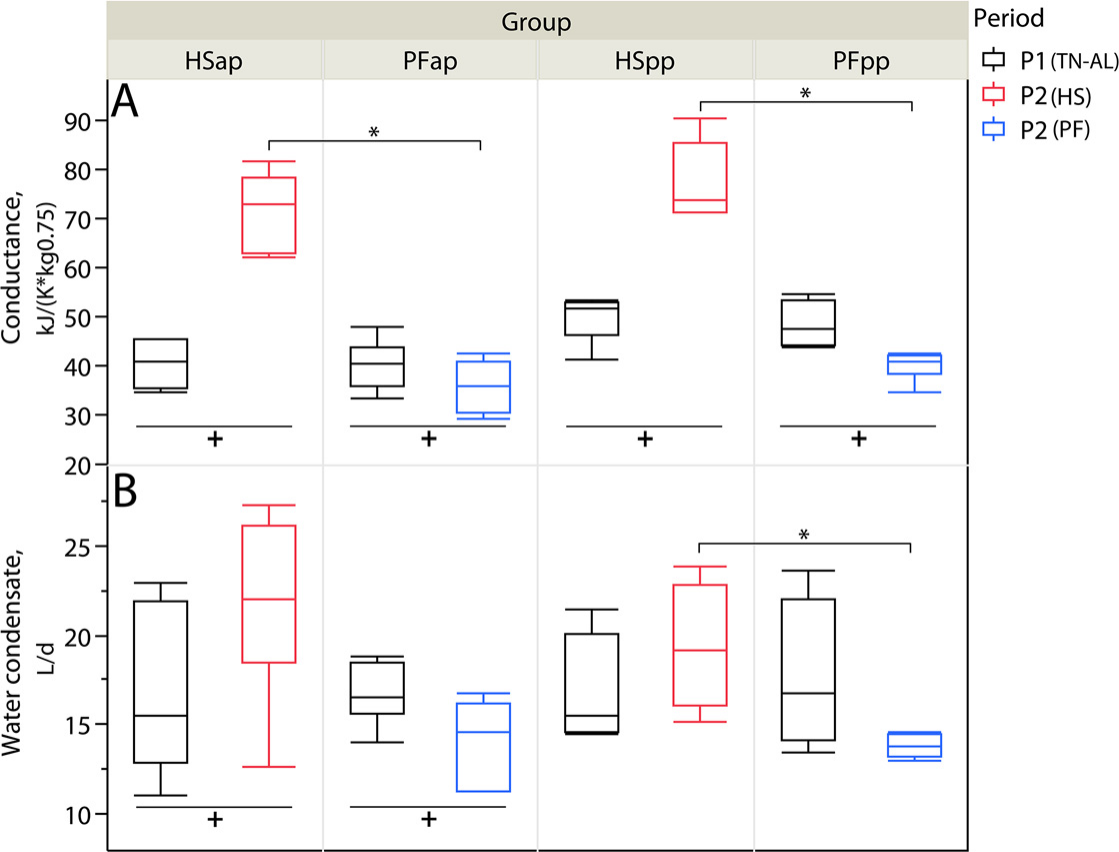
Conductance and Water Condensate. Effect of heat stress (HS) and pair-feeding (PF) on (A) conductance and (B) water condensate. In experimental period 1 (P1, black) all animals were kept at thermoneutral conditions (TN, THI = 59.7) with ad-libitum feeding (AL) for six days. During period 2 (P2), HS cows (red) were heat-stressed (THI = 76.1), whereas PF cows (blue) were pair-fed in thermoneutrality (THI = 60.0) for six days, once ante partum (ap) and again post-partum (pp). Data acquired during the 24-hour indirect calorimetry analysis on day 5 of each period. (A) For conductance analysis, numbers of animals analyzed per group: HSap n = 7, PFap n = 6, HSpp n = 6, PFpp n = 6; (B) For water condensate measurement, numbers of animals analyzed per group: HSap n =, 6 PFap n = 6, HSpp n = 4, PFpp n = 4; ^**+**^P < 0.05 in Wilcoxon signed rank sum test for paired samples of the same group; there were no differences in groups of the same reproductive stage in P1 (P > 0.05) according to the exact Wilcoxon-Mann-Whitney test; ^*^P < 0.05 in the exact Wilcoxon-Mann-Whitney test for group differences of the same reproductive stage in P2.

The conductance of HS animals strongly increased during P2 (P<0.05), whereas, the conductance of PF cows slightly decreased under feed restriction of P2 (P<0.05, Fig 5A). In the ap stage, water condensate as a measure of evaporative water emission was increased under HS conditions, but reduced under PF feed restriction (each P<0.05, Fig 5B). However, in the pp stage, HS cows produced more water condensate during P2 than PF cows (P<0.05), while the volumes did not differ in P1 (Fig 5B).

### Plasma 1-/3-methyl histidine concentrations are elevated in late-gestating cows under HS conditions

In HSap cows, we observed an increase in plasma 1-/3-methyl histidine concentrations from P1 to P2 (P<0.05), resulting in higher concentrations than in PFap animals during P2 (P<0.05). However, in all other groups methyl histidine concentrations remained constant (Table 4).

**Table 4:** Plasma 1-/3-methyl histidine concentrations of heat-stressed (HS) and pair-fed (PF) cows on day 6 of each period.

		Period		
Item	Group	P1^1^	P2^1^	P-Value^2^
1-/3-methyl histidine, μmol/L	HSap	7.7 [5.6; 9.2]^a^	12.2 [10.1; 12.8]^a^	0.031
	PFap	7.0 [3.5; 10.7]^a^	7.8 [6.4; 10.9]^b^	0.438
	HSpp	9.3 [5; 22]^c^	11.7 [8.3; 14.5]^c^	0.438
	PFpp	6.4 [4.2; 8.3]^c^	9.3 [4.3; 23.2]^c^	0.438

ap ante partum; pp post-partum; P1 period of thermoneutral conditions (THI = 59.7) with ad libitum feeding; P2 period of either pair feeding at thermoneutral (THI = 60.0) conditions (PF) or during ad libitum feeding under heat exposure (HS, THI = 76.1).

Numbers of animals analyzed per group: HSap n = 7, PF ap n = 6, HS pp n = 6, PF pp n = 6.

^1^ Values are given as mean [minimum; maximum].

^2^ P-Values derived from Wilcoxon signed-rank test for paired samples.

^a,b^Values with differing superscripts denote group differences within period (column) and ap stage (P < 0.05, based on the exact Mann-Whitney U-test for independent samples).

^c,d^Values with differing superscripts denote group differences within period (column) and pp stage (P < 0.05, based on the exact Mann-Whitney U-test for independent samples).

### Heat stress induces a strong adrenergic response in both reproductive stages

**Fig 6:**
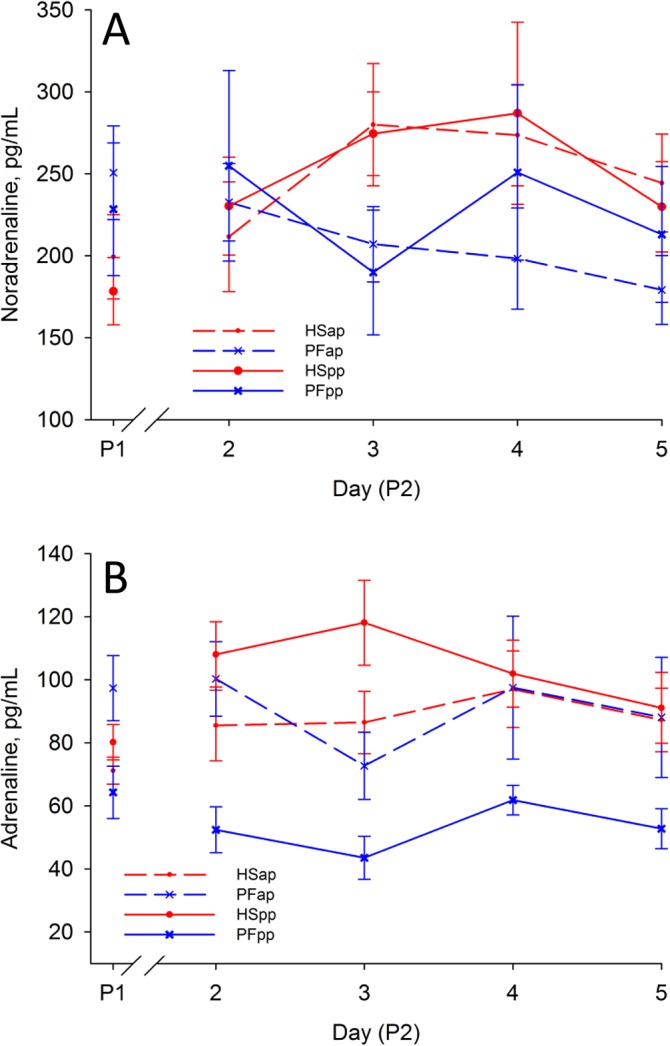
Catecholamines. Effect of heat stress (HS) and pair-feeding (PF) on (A) plasma noradrenaline and (B) adrenaline. In experimental period 1 (P1) all animals were kept at thermoneutral conditions (THI = 59.7) with ad-libitum feeding for six days. During period 2 (P2), HS cows (red lines) were heat-stressed (THI = 76.1), whereas PF cows (blue lines) were pair-fed in thermoneutrality (THI = 60.0) for six days, once ante partum (ap, dashed lines) and again post-partum (pp, solid lines). All data given as mean ± SEM; for P1 the mean of the last days of period 1 is given as used for baseline correction. Numbers of animals analyzed per group: HSap n = 7, PFap n = 6, HSpp n = 6, PFpp n = 6.

Only in the ap stage, noradrenaline concentrations were increased due to heat stress conditions, whereas feed restriction alone lowered noradrenaline plasma concentrations (group: P < 0.05, Fig 6A, Table 3). In contrast, lactating cows exhibited marked reactions in adrenaline concentrations: While plasma adrenaline increased in HSpp cows, it was almost unaffected in PFpp (group: P < 0.05). Late-gestating cows showed no significant adrenaline reaction in P2 (Fig 6B, Table 3).

## Discussion

Recently it has been postulated that heat-stressed dairy cows in established lactation alter their metabolic and fuel selection priorities independent of nutrient intake or energy balance [12]. These shifts in nutrient partitioning involve changes in post absorptive lipid, carbohydrate, and amino acid utilization. The aim of this study was to evaluate these effects of heat stress on macronutrient and energy metabolism in late-gestating and early-lactating Holstein cows using indirect calorimetry combined with plasma metabolite analyses. In the present study dairy cows of both reproductive stages responded to a sudden rise of ambient temperatures with a marked decrease in DMI, higher respiratory rates and rectal temperatures as well as increased catecholamine plasma concentrations which all were lacking in the PF controls. The THI and the duration of the challenge were comparable to conditions described earlier for mid-lactating cows [12–14]. Accordingly, the mean increase in rectal temperatures of heat-stressed lactating cows of 3.1% was similar to the increments observed in earlier studies of 3.08% and 3.16%, respectively [12, 14]. But it was lower than the mean increase of rectal temperatures of 4.08% reported by Wheelock et al. [13], although the mean rectal temperature of our HSpp animals (40.09°C) was slightly higher than the mean rectal temperature during heat stress (40.00°C) given in that earlier study [13]. Overall, rectal temperatures of late-gestating HS cows only increased by 1.84% due to an endogenic heat production lower than in lactating cows.

The mean daily milk yield of both groups during the last three days of thermoneutral P1, clearly exceeding the 30 liter threshold (Fig 1B), was equal or even higher than those reported in the earlier studies mentioned above [12–14], like the HS-induced relative reduction in milk yield which lay between the reductions described earlier [12–14]. However, in the present study HS and PF cows in early lactation did not differ in milk loss. It seems that our results confirm the old idea that heat stress-induced reduction in milk yield is primarily a consequence of decreased feed intake [8–10,28], which would contradict more recent reports [12,13]. Here it has to be taken into account that the prioritization of milk synthesis is higher in early than in mid lactation examined as indicated by the typical rise of milk production during the first weeks of lactation [5,15]. This might lead to a neutralization of the additional, heat stress-related decrease in milk synthesis described previously [12,13] resulting in the same decrement of milk yield in HS and PF in early lactation. In the present study design, carryover effects could have confounded the performance of the postpartal group as it was described for cows heat-stressed in the dry period [29,30]. However, we found no differences in milk yield between both groups during the thermoneutral, ad libitum feeding period, arguing against a carryover effect from the ap to the pp period in our experiment. Differences observed between lactating HS and PF animals during P1 were only found for respiratory rate being slightly lower in HSpp.

### Heat production, carbohydrate and fat metabolism

As determined by indirect calorimetry, HS and PF reduced metabolic heat production to the same extent in both, late-gestating and early-lactating dairy cows. This effect may be explained by the same decline of feed intake in both groups which is in accordance with earlier experiments [31]. Feed of ruminants is primarily composed of carbohydrates, and therefore the reduction of feed intake during HS and PF resulted in a reduced COX in all groups, despite this effect was only numerical for HSap animals (Fig 4B). Considering, that the oxidation of 1 g carbohydrate provides 17 kJ, the average reduction of COX/mBW by 10 g/(d*kg^0.75^) from ad libitum feeding to HS or PF contributes to mitigate endogenous HP by 170 kJ/(d*kg^0.75^), which explains the reduction in HP to a major extent. However, the contributions of fat and carbohydrate oxidation to heat production are differently pronounced in HS and PF animals. Whereas under PF conditions FOX/mBW (Fig 4C) and plasma NEFA (Fig 2D) concentrations increased, they were not altered in heat-stressed early-lactating cows. Thus, the basal level of adipose tissue mobilization in early lactation is not suppressed but also not increased by heat stress, which adds to earlier findings that heat stress prevents an increase in plasma NEFA concentrations of mid-lactating dairy cows at negative energy balance [12,13,32]. Our results provide further novel evidence that non-responding fatty acid mobilization from adipose tissue during heat stress is paralleled by an unchanged FOX/mBW in early lactation, despite the reduction of feed intake. Thus it is likely that HS cows, in contrast to PF animals, dispense on increasing FOX as a strategy to prevent enhanced mitochondrial substrate oxidation, endogenous heat production and formation of reactive oxygen species, a serious adverse effect of heat stress [33,34], as proposed earlier [35,36]. Moreover, the decreased COX/FOX ratio further indicates a shift towards fat oxidation after feed restriction at 15°C, while both indicators were not altered under heat stress conditions. The dependence on carbohydrate utilization as the major source of energy during heat stress is also reflected by the lacking decrease of COX/mBW in HSap cows. Therefore, our findings support the hypothesis of a preference for carbohydrate over lipid oxidation under heat stress conditions [12,37]. The preferential use of glucose as the major energy substrate in peripheral tissues is at the expense of milk production in heat stressed mid-lactation cows, [12–14] and our results show that this is also true for early-lactating cows.

Notably, in contrast to lactating cows, late-gestating animals showed a strong increase of plasma NEFA concentration in response to heat exposure while FOX/mBW did not change simultaneously. These results confirm earlier observations that heat stress in late gestation led to an increase of circulating NEFA concentrations [30,38–40] but contrast the recent assumption that the coordinated systemic response to heat stress is conserved across physiological states [35].

The increase of plasma NEFA and the decrease of glucose concentrations in heat-stressed dry cows at a maintained FOX/mBW and COX/mBW levels suggest a higher heat tolerance of non-lactating cows [1]. This is probably due to their lower metabolic heat production as the energy demand of late gestation equals a milk output of only 3 to 6 kg milk/day [15] reflected in the lower heat load during HS described by lower rectal temperatures than in lactating HS cows. Thus, the anti-lipolytic effect of heat stress as observed in the HSpp group and described previously [12,13,32] does not occur in late-pregnant dry cows. However, mobilized NEFA are not completely oxidized, but possible consumers of NEFA may be the developing mammary gland which is increasing its alveolar gland tissue during lactogenesis with a demand for membrane lipids [15] and the uterus. Greater amounts of NEFA might be taken up by the placenta as shown by increased abundance of fatty acid transporter mRNA in cotyledons in nutrient-restricted mid-gestation ewes [41]. But so far, utilization of NEFA by the fetus has been assessed to be of minor relevance in the fetal calf [42]. The bovine fetus is mainly dependent on oxidation or anaerobic glycolysis of carbohydrates like glucose, lactate and amino acids [15,42]. In heat stress, accumulation of the general fetal carbohydrate preference and the enhanced maternal prioritization of carbohydrate oxidation correspond to the maintenance of COX/mBW and higher plasma glucose concentrations in HSap cows.

Still, further research is required to elucidate the utilization of macronutrients and in particular of fatty acids in heat-stressed, nonlactating, pregnant cows.

### Protein metabolism

As DMI declines during heat stress and glycogen reservoirs are limited in times of energy scarcity [11], carbohydrate delivery to the consuming tissues has to be maintained by gluconeogenesis from endogenous sources such as amino acids. In this situation, an increase of amino acid utilization for energy supply leads to a higher urea synthesis as well as higher methyl histidine concentrations as a marker of muscle fiber proteolysis [43,44]. In the present study, we could detect increments in plasma urea exclusively in early-lactating cows (Fig 3A), and these increments were stronger in heat-stressed than in PF animals. For lactating cows in heat stress, several studies [13,32,34,45] have also described higher plasma urea-N concentrations, whereas the plasma urea-N concentrations of cows cooled in the dry period did not differ from uncooled controls [30,38].

Different factors cause an increase of plasma urea concentrations including higher hepatic urea production as well as lowered renal clearance as a consequence of impaired kidney perfusion. Under acute heat stress, reduced kidney perfusion is a result of sudden temperature-associated vasodilation in peripheral body layers and higher evaporative water loss leading to a lowered blood pressure [46] and subsequently enhanced water retention [47]. In agreement with these reports, we observed a reduced hematocrit (Fig 2A) or an increased circulating plasma fraction, respectively, as a heat stress-related reaction usually observed in humans [48]. Apparently, our results are in contrast to the Obitsu study [45] stating that renal clearance after 9 to 14 days under high ambient temperatures is unaffected. However, in the present study we investigated only the first 6 days of a heat stress period, where impairments of biological functions may occur before homeostasis is recovered [7].

To examine the reasons for uremia in challenged early-lactating cows, we analyzed plasma creatinine as a constantly produced marker of renal clearance [49]. In the bovine as in many other species, creatinine synthesis is an individual constant, which depends on muscle mass [50], but not on diet [50–52]. Therefore, the increased plasma creatinine concentrations observed in the present and earlier studies [34,45,53], might be mainly caused by a reduction in renal clearance and only in part by an increased acute muscle catabolism [54].

The latter is also characterized by increased plasma methyl histidine concentrations [54], although recent studies described only a minor increase of methyl histidine in response to heat stress [43]. Therefore, increased plasma methyl histidine accompanied by increased circulating creatinine indicates strong muscle proteolysis in the HSap group.

To eliminate the effect the effect of renal insufficiency on altered plasma urea and creatinine concentrations, we calculated the urea/creatinine ratio [55] assessing the balance between protein oxidation and proteolysis. As this ratio remained unchanged for the HSpp group (Fig 3C), it seems that uremia in this group is mainly caused by a temporary renal insufficiency during acute acclimation affecting urea and creatinine clearance identically. In contrast, in proteolytic HSap cows, the declining urea/creatinine ratio point rather towards a different pattern of protein utilization. It seems that in HSap cows, amino acid mobilization from muscle protein reserves exceeds amino acid oxidation in the liver and that mobilized amino acids are directed to the fetus, thereby maintaining the high growth rate during the last weeks of fetal development [56] or as an alternative fetal fuel as stated above.

The opposite occurs in non-gestating cows. Insufficient energy intake is mainly encountered by a reduction of milk production, and at least in part compensated by a higher rate of protein oxidation leading to higher plasma urea concentrations in PFpp and HSpp. In HSpp, the constant urea/creatinine ratio reflects a relative equilibrium of muscle proteolysis contributing to the plasma creatinine increment on the one hand and urea synthesis from oxidized amino acids on the other.

The different utilization of amino acids depending on the reproductive stage, calls for an adequate dietary protein supply during hot weather conditions. However, precise requirements and recommendations need to be elaborated in future studies for lactating and gestating cows, separately.

### Water homeorhesis

High ambient temperatures increase water requirements for evaporative cooling, and, as described in the previous section, water retention is enhanced to maintain the blood pressure under heat-induced vasodilation [47]. As expected, we observed higher evaporative water losses in both HS groups than in the PF controls (Fig 5B) which are similar to earlier reports on increased sweating [32,57,58]. As a conclusion, the newly presented method of exhaust air humidity condensation allows a simple and robust estimate of evaporative water loss in open-circuit respiration chambers. Moreover, evaporative water losses are paralleled by an increased conductance in both HS groups while they decrease in the controls, which is similar in mice kept under varying ambient temperatures [26]. We also found enhanced water retention reflected in lowered hematocrit values of HS cows as described above. Higher water loss and retention requires a corresponding drinking behavior which we detected as an increased water intake per kg DMI in both HS groups (Fig 1F). However, the water intake normalized to DMI in our study was higher than recently reported by Khelil-Arfa et al. [59] which might be due to the higher feed DM content used in the present study. Thus, the six-day heat stress applied in the present study causes marked effects on water homeorhesis on the level of intake, retention and evaporative loss.

### Catecholaminergic response

Heat stress is an inductor of catecholamine release in the bovine [60].While pair-feeding during P2 caused no changes or even a decline of catecholamine concentrations, high ambient temperatures increased adrenaline in lactating and increased noradrenaline in dry cows (Fig 6). Catecholamines are major inductors of lipolysis and glucose provision [12,14,61], thus it is initially surprising that highest levels of catecholamines are observed in heat-stressed early-lactating cows combined with constant NEFA concentrations and lowered glucose concentrations. Nevertheless, our findings are in line with the results described by Baumgard et al. [14] showing that an epinephrine challenge increases plasma NEFA concentrations in mid-lactating PF cows while this response was lacking in HS cows. Apparently, the high glucose concentrations during Baumgard’s epinephrine challenge are in contrast to the low plasma glucose found in our experiment, but can be explained by the different experimental approaches. As the epinephrine challenge uses an artificial, short-term adrenaline impact inducing hyperglycemia and a delayed increase of glucose clearance, we have observed a homeorhetic system over a period of 6 days in which glucose provision and utilization are balanced. Following the aforementioned theory of preferential carbohydrate oxidation in heat-stressed cows, the enhanced adrenergic response is a logical step to increase hepatic glycogenolyis and gluconeogenesis whereas adipose tissue becomes refractory to lipolytic signals in heat-stressed lactating cows as described by Baumgard et al. [14].

## Conclusions

The objective of the present study was to compare the macronutrient oxidation in late-gestating versus early-lactating dairy cows fed either ad libitum during high ambient temperatures or pair-fed at thermoneutrality. Earlier studies utilizing the pair-feeding approach [12–14] focused on mid-lactating cows which are highly susceptible to heat stress at their maximum of milk production. In contrast, our focus was set on the transition cow, because the transition phase is not only a vulnerable period, but highly determinative for the subsequent lactation. To gain novel insights into the oxidative metabolism of different macronutrients during heat stress and pair-feeding beyond the level of metabolite analysis, we applied the technique of indirect calorimetry. We could demonstrate that fat oxidation is not affected by heat-induced reduction of feed intake in both, late-gestating and early-lactating cows despite a different lipolytic response. Moreover, cows under high ambient temperatures exhibited a metabolic shift towards a pronounced carbohydrate oxidation. To provide a sufficient supply of carbohydrate precursors under hot thermal conditions, these cows extensively degrade tissue protein reflected in the response of plasma urea, creatinine and methyl histidine concentrations. However, the acute metabolic heat response in late-gestating dry cows seems to differ from early-lactating cows as the prepartal adipose tissue is not refractory to lipolytic signals which were present in both stages and the degree of amino acid oxidation may be lower than in the postpartal situation. Both differences might be attributed to nutrient requirements of the fast-growing, near-term fetus and to a higher heat tolerance due to a lower endogenous metabolic heat load in dry cows. It might be useful to consider these divergent demands in the development of nutritional strategies for the attenuation of heat stress impairments of dairy health and performance in the future.

## Supporting Information

S1 Fig Baseline-corrected Nutritional and Vital Data. In experimental period 1 (P1) all animals were kept at thermoneutral conditions (THI = 59.7) with ad-libitum feeding for six days. During period 2 (P2), HS cows (red lines) were heat-stressed (THI = 76.1), whereas PF cows (blue lines) were pair-fed in thermoneutrality (THI = 60.0) for six days, once ante partum (ap, dashed lines) and again post-partum (pp, solid lines). All data given as LSMeans ± SEM. THI Temperature humidity index; DMI dry matter intake. Results of Tukey-Kramer test (HS vs. PF on same day): ap P < 0.05 marked with asterisk; pp P < 0.05 marked with bold cross.
(TIF)

S2 Fig Baseline-corrected Hematocrit and Plasma Metabolites. In experimental period 1 (P1) all animals were kept at thermoneutral conditions (THI = 59.7) with ad-libitum feeding for six days. During period 2 (P2), HS cows (red lines) were heat-stressed (THI = 76.1), whereas PF cows (blue lines) were pair-fed in thermoneutrality (THI = 60.0) for six days, once ante partum (ap, dashed lines) and again post-partum (pp, solid lines). All data given as LSMeans ± SEM. Results of Tukey-Kramer test (HS vs. PF on same day): pp P < 0.05 marked with bold cross, 0.05 ≤ P < 0.07 marked with thin cross.
(TIF)

S3 Fig Baseline-corrected Plasma Urea and Creatinine. In experimental period 1 (P1) all animals were kept at thermoneutral conditions (THI = 59.7) with ad-libitum feeding for six days. During period 2 (P2), HS cows (red lines) were heat-stressed (THI = 76.1), whereas PF cows (blue lines) were pair-fed in thermoneutrality (THI = 60.0) for six days, once ante partum (ap, dashed lines) and again post-partum (pp, solid lines). All data given as LSMeans ± SEM. Results of Tukey-Kramer test (HS vs. PF on same day): ap P < 0.05 marked with asterisk; pp P < 0.05 marked with bold cross.
(TIF)

S4 Fig Baseline-corrected Plasma Catecholamines. In experimental period 1 (P1) all animals were kept at thermoneutral conditions (THI = 59.7) with ad-libitum feeding for six days. During period 2 (P2), HS cows (red lines) were heat-stressed (THI = 76.1), whereas PF cows (blue lines) were pair-fed in thermoneutrality (THI = 60.0) for six days, once ante partum (ap, dashed lines) and again post-partum (pp, solid lines). All data given as LSMeans ± SEM. Results of Tukey-Kramer test (HS vs. PF on same day): pp P < 0.05 marked with bold cross.
(TIF)

S1 Table Complete dataset of variables analyzed during indirect calorimetry on day 6 of periods 1 and 2. HS heat stress group; PF pair-fed group; ap ante partum; pp post-partum; P1 period 1 (thermoneutral conditions, ad libitum feeding); P2 period 2 (either heat stress, ad libitum feeding (HS) or thermoneutral conditions, pair-feeding(PF)); Genotype of HSP70.1 5′-UTR 895 according to [16]; BW body weight (kg); BFT back fat thickness (mm); HP/mBW heat production per metabolic weight (kJ/d*kg^0.75^); COX/mBW net carbohydrate oxidation per metabolic weight (g/(d*kg^0.75^)); FOX/mBW net fat oxidation per metabolic weight (g/d* kg^0.75^); EB energy balance (MJ); Water_cond water condensate (L/d); Conductance (kJ/(K*kg^0.75^)).
(XLS)

S2 Table Complete dataset of all variables analyzed continuously during periods 1 and 2. HS heat stress group; PF pair-fed group; ap ante partum; pp post-partum; P1 period 1 (thermoneutral conditions, ad libitum feeding, given as mean of the last days of P1 used for baseline correction); P2 period 2 (either heat stress, ad libitum feeding (HS) or thermoneutral conditions, pair-feeding(PF), given for day 1 to day 6); Hematocrit (L/L); Glucose (mmol/L); BHBA β-hydroxybutyric acid (mmol/L), NEFA non-esterified fatty acids (μmol/L); Urea (mmol/L); Creatinine (μmol/L); Urea/Creatinine ratio (mmol/μmol); NA noradrenaline (pg/ml); A adrenaline (pg/ml); RT rectal temperature (°C); RR respiratory rate (counts/min); HR heart rate (counts/min); DMI dry matter intake (kg); Water intake/DMI (L/kg); Milk yield (L).
(XLS)

S3 Table Complete dataset of Temperature Humidity Index (THI) and Dry Matter Intake (DMI) during Period 1 (P1) and Period 2 (P2). (XLS)
