# Supplementary figures and images for: Metabolic Heat Stress Adaption in Transition Cows: Differences in Macronutrient Oxidation between Late-Gestating and Early-Lactating German Holstein Dairy Cows
Source: PLoS One. 2015 May 4;10(5):e0125264. doi: 10.1371/journal.pone.0125264 (PMC4418699; doi:10.1371/journal.pone.0125264)

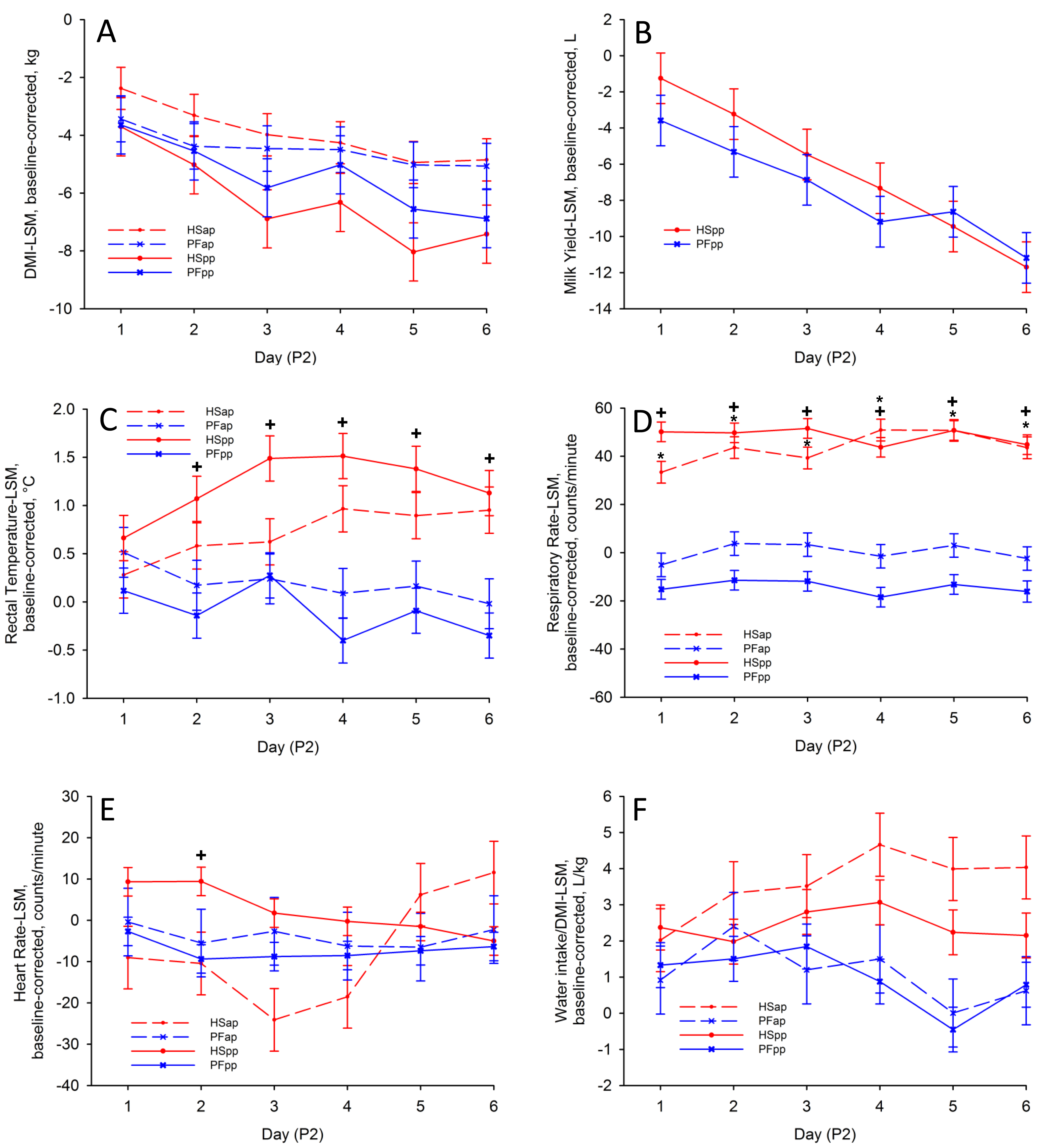

Supplement: S1 Fig — In experimental period 1 (P1) all animals were kept at thermoneutral conditions (THI = 59.7) with ad-libitum feeding for six days. During period 2 (P2), HS cows (red lines) were heat-stressed (THI = 76.1), whereas PF cows (blue lines) were pair-fed in thermoneutrality (THI = 60.0) for six days, once ante partum (ap, dashed lines) and again post-partum (pp, solid lines). All data given as LSMeans ± SEM. THI Temperature humidity index; DMI dry matter intake. Results of Tukey-Kramer test (HS vs. PF on same day): ap P < 0.05 marked with asterisk; pp P < 0.05 marked with bold cross. (TIF) [file pone.0125264.s001.tif]

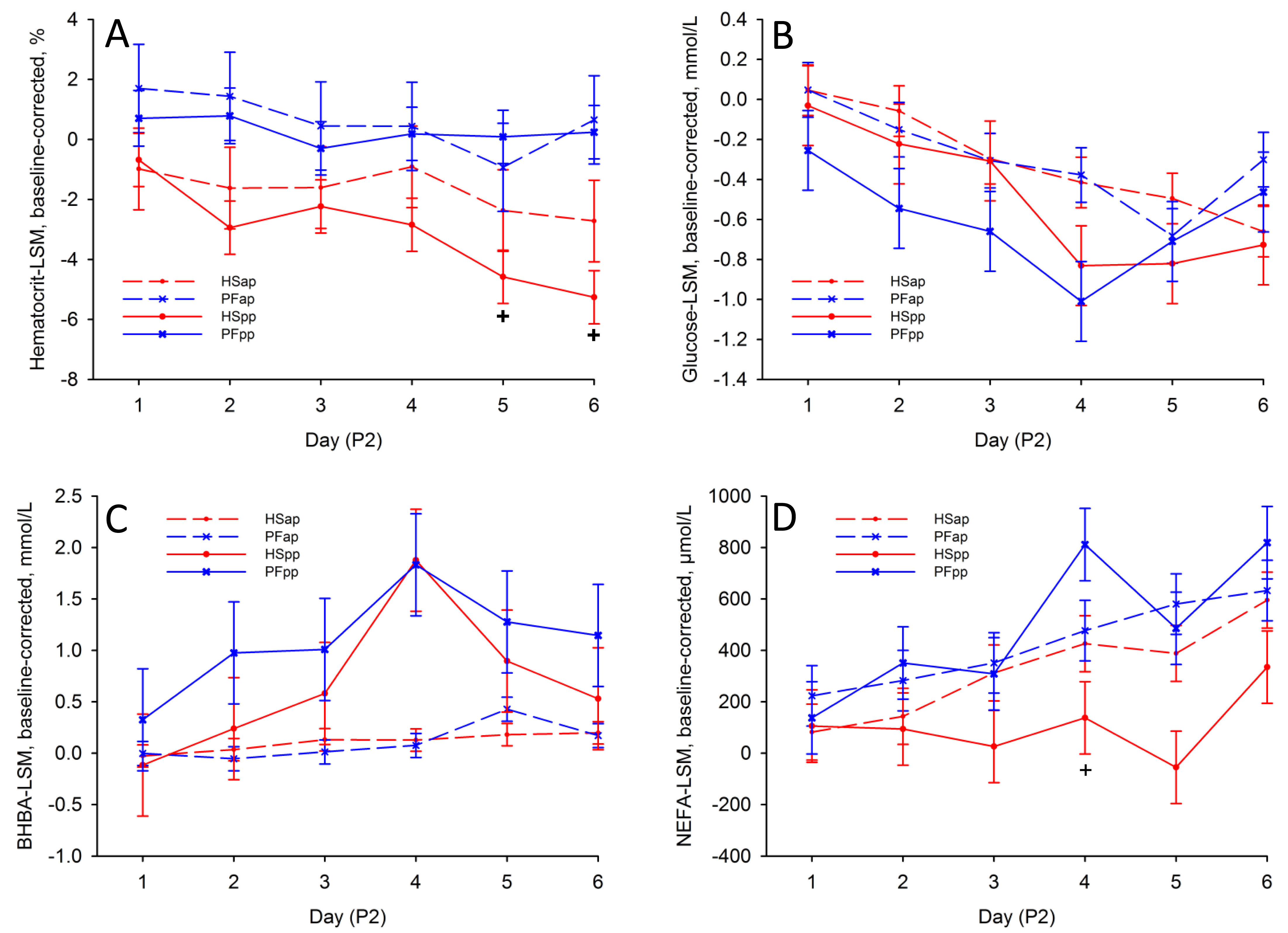

Supplement: S2 Fig — In experimental period 1 (P1) all animals were kept at thermoneutral conditions (THI = 59.7) with ad-libitum feeding for six days. During period 2 (P2), HS cows (red lines) were heat-stressed (THI = 76.1), whereas PF cows (blue lines) were pair-fed in thermoneutrality (THI = 60.0) for six days, once ante partum (ap, dashed lines) and again post-partum (pp, solid lines). All data given as LSMeans ± SEM. Results of Tukey-Kramer test (HS vs. PF on same day): pp P < 0.05 marked with bold cross, 0.05 ≤ P < 0.07 marked with thin cross. (TIF) [file pone.0125264.s002.tif]

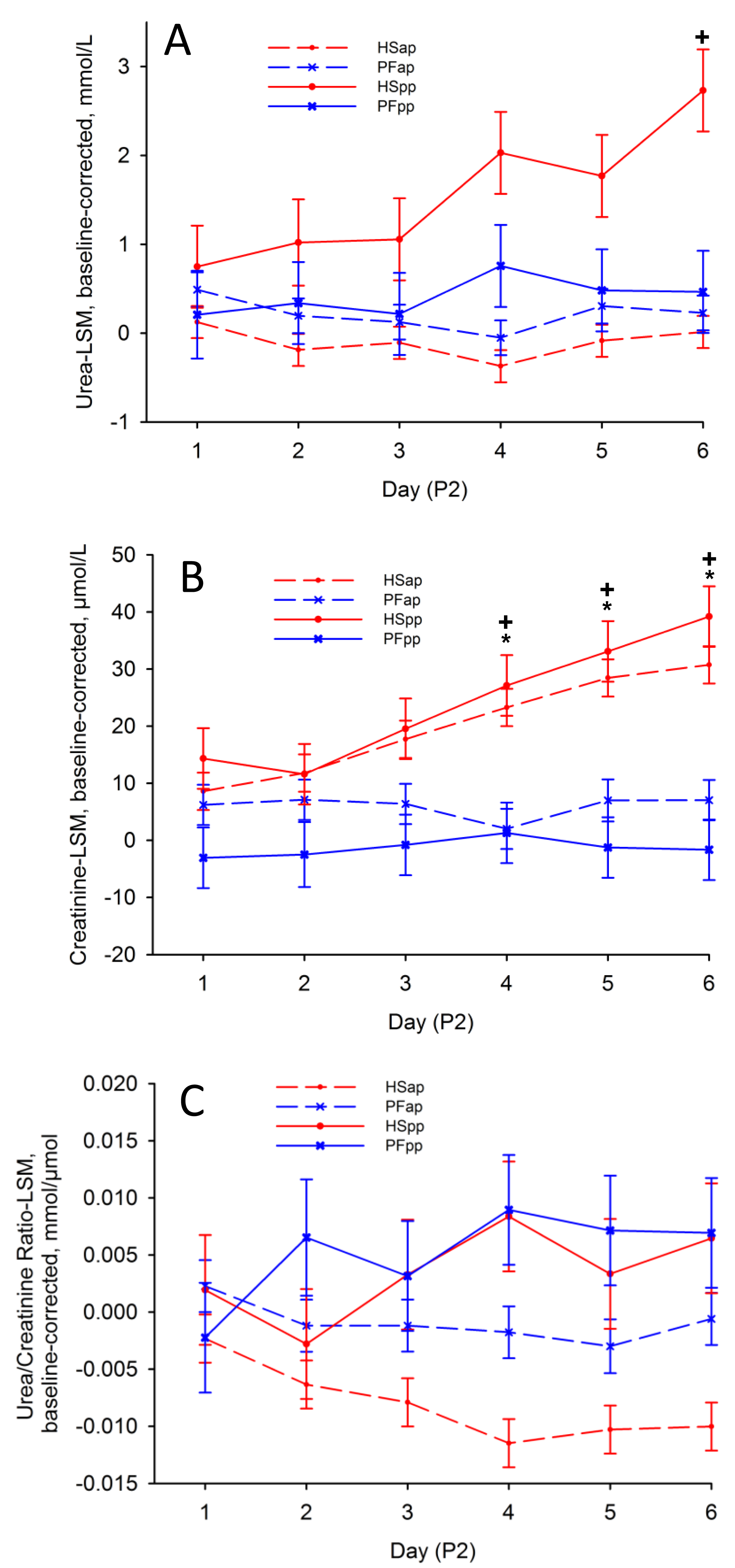

Supplement: S3 Fig — In experimental period 1 (P1) all animals were kept at thermoneutral conditions (THI = 59.7) with ad-libitum feeding for six days. During period 2 (P2), HS cows (red lines) were heat-stressed (THI = 76.1), whereas PF cows (blue lines) were pair-fed in thermoneutrality (THI = 60.0) for six days, once ante partum (ap, dashed lines) and again post-partum (pp, solid lines). All data given as LSMeans ± SEM. Results of Tukey-Kramer test (HS vs. PF on same day): ap P < 0.05 marked with asterisk; pp P < 0.05 marked with bold cross. (TIF) [file pone.0125264.s003.tif]

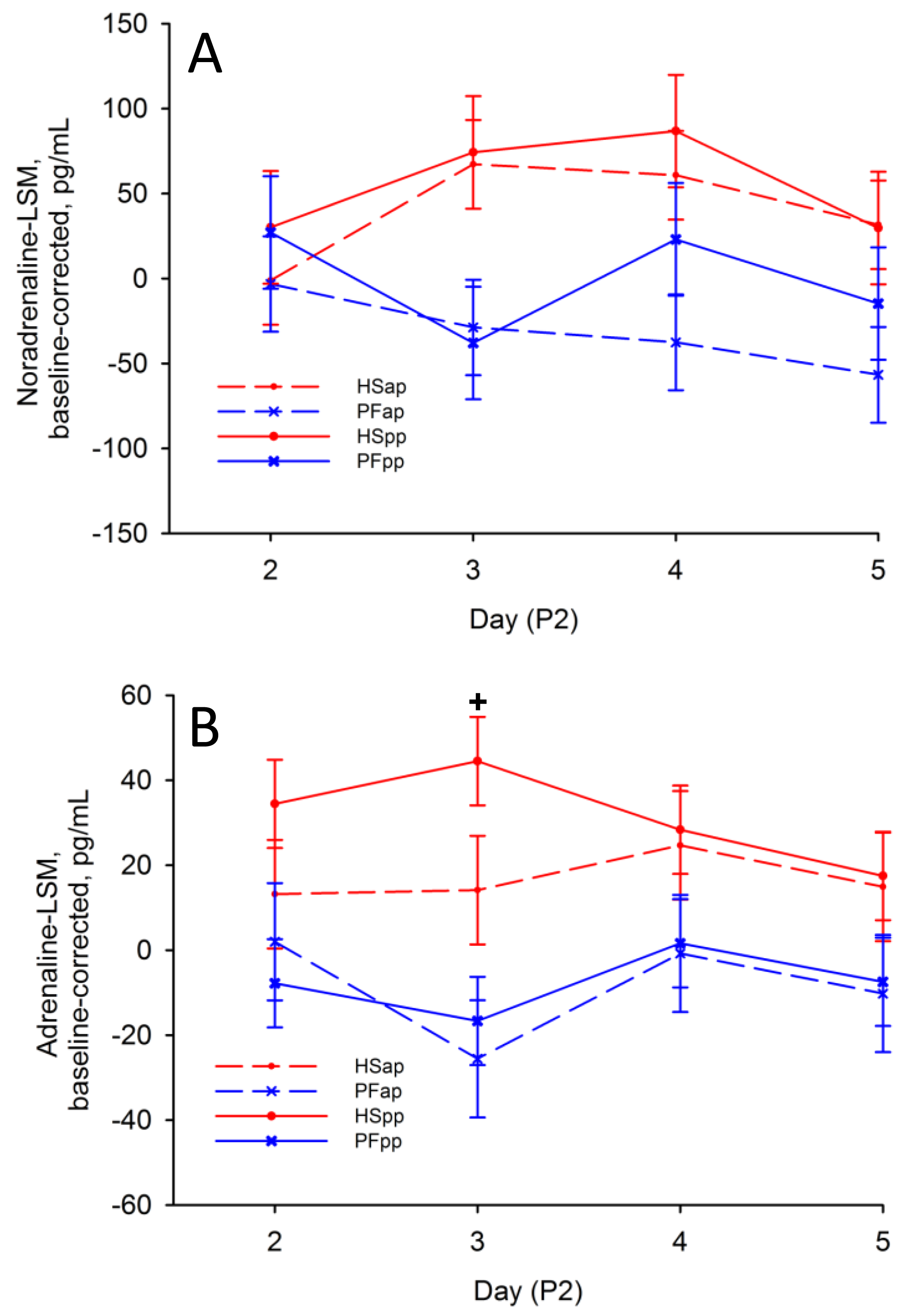

Supplement: S4 Fig — In experimental period 1 (P1) all animals were kept at thermoneutral conditions (THI = 59.7) with ad-libitum feeding for six days. During period 2 (P2), HS cows (red lines) were heat-stressed (THI = 76.1), whereas PF cows (blue lines) were pair-fed in thermoneutrality (THI = 60.0) for six days, once ante partum (ap, dashed lines) and again post-partum (pp, solid lines). All data given as LSMeans ± SEM. Results of Tukey-Kramer test (HS vs. PF on same day): pp P < 0.05 marked with bold cross. (TIF) [file pone.0125264.s004.tif]
